# circPRRC2A promotes angiogenesis and metastasis through epithelial-mesenchymal transition and upregulates TRPM3 in renal cell carcinoma

**DOI:** 10.7150/thno.43239

**Published:** 2020-03-15

**Authors:** Wei Li, Feng-Qiang Yang, Chen-Min Sun, Jian-Hua Huang, Hai-Min Zhang, Xue Li, Guang-Chun Wang, Ning Zhang, Jian-Ping Che, Wen-Tao Zhang, Yang Yan, Xu-Dong Yao, Bo Peng, Jun-Hua Zheng, Min Liu

**Affiliations:** 1 Department of Urology, Shanghai Tenth People's Hospital, Tongji University, Shanghai, P. R. China.; 2 Department of Medical Oncology, Dana-Farber Cancer Institute, Harvard Medical School, Boston, MA, USA.; 3 Department of Urology, Ninghai Hospital, Branch of Shanghai Tenth People's Hospital, Zhejiang, P. R. China.; 4 Department of Anesthesiology, Tongren Hospital, Shanghai JiaoTong University School of Medicine, Shanghai, P. R. China.; 5 Department of Pathology, Beijing Chao-Yang Hospital, Capital Medical University, Beijing, P. R. China.; 6 Department of Urology, Shanghai First People's Hospital, Shanghai JiaoTong University School of Medicine, Shanghai, P. R. China.; 7 Department of Urology, Tongren Hospital, Shanghai JiaoTong University School of Medicine, Shanghai, P. R. China.

**Keywords:** circular RNA, RCC, circPRRC2A, lung metastasis, therapeutic target

## Abstract

**Background**: Circular RNAs (circRNAs) have been identified as essential regulators in a plethora of cancers. Nonetheless, the mechanistic functions of circRNAs in Renal Cell Carcinoma (RCC) remain largely unknown.

**Methods**: In this study, we aimed to identify novel circRNAs that regulate RCC epithelial-mesenchymal transition (EMT), and to subsequently determine their regulatory mechanisms and clinical significance.

**Results**: circPRRC2A was identified by circRNA microarray and validated by qRT-PCR. The role of circPRRC2A in RCC metastasis was evaluated both *in vitro* and *in vivo*. We found that increased expression of circPRRC2A is positively associated with advanced clinical stage and worse survivorship in RCC patients. Mechanistically, our results indicate that circPRRC2A prevents the degradation of TRPM3, a tissue-specific oncogene, mRNA by sponging miR-514a-5p and miR-6776-5p. Moreover, circPRRC2A promotes tumor EMT and aggressiveness in patients with RCC.

**Conclusions**: These findings infer the exciting possibility that circPRRC2A may be exploited as a therapeutic and prognostic target for RCC patients.

## Introduction

In the last several decades, there has been a progressive increase in the incidence of renal cell carcinoma (RCC) in Asia, the United States and Europe, reaching about 3% per year and causing ~90,000 deaths worldwide annually 1. Thirty percent of patients had metastatic lesions at initial diagnosis, and greater than 30% ultimately developed metastatic renal cell carcinoma (mRCC) post operation 2,3. In cases of RCC that have become metastatic, available therapies often fail to effectively reduce tumor growth or achieve remission. These treatments have a response rate of between 15% and 25%, and subsequently the overall median survival of patients with mRCC is less than 1 year 4. Early detection of metastatic RCC is likely to improve the capability to predict patient prognosis and could be used to discern which patient cohorts would benefit from specific targeted therapies 5. Unfortunately, unlike gastrointestinal tumors, biomarkers for early diagnosis and monitoring of RCC are unavailable at present 6. To devise therapeutic strategies with greater efficacy, it is crucial to decipher the molecular mechanisms involved in the development and progression of RCC.

Circular RNAs (circRNAs) are a new class of endogenous RNAs characterized by covalently closed loops formed by back splicing events. While circRNAs are widely expressed in mammals, unlike mRNA, they do not have 5' to 3' polarity or polyadenylated tails 7-11. While linear RNAs are degraded by RNase R digestion, circRNAs are not 12,13. Accumulating evidence has established that circRNAs perform vital roles in the pathogenesis of human diseases, especially in malignant tumors, such as triple- negative breast cancer, lung adenocarcinoma, colon cancer and hepatocellular carcinoma 14,15. To date, a few studies have reported that circRNAs compete for the binding of miRNAs, and therefore have been termed competing endogenous RNAs (ceRNAs), in several types of cancer 16-18. Zhou *et al.* showed that cSMARCA5 (sponge of miR-17-3p/miR-181b-5p) suppresses the proliferation and migration of hepatocellular carcinoma, suggesting cSMARCA5 as a potential therapeutic target 19,20. However, the expression features, function and potential molecular mechanisms of novel identified circRNAs in RCC warrant further study.

While studies into the dysregulation of circRNAs and their possible involvement in RCC are still rare, several circRNAs have been identified to be significantly dysregulated in RCC. Chang *et al.* found a novel circHIAT1 RNA that was lower in expression in RCC than tumor adjacent tissue. Forced expression of circHIAT1 lead to a suppression of AR-enhanced RCC proliferation and invasion 21. While circRNAs have been found to be critical for RCC progression, the functioning of circRNAs in RCC are still unclear. Wang *et al.* investigated the expression profile of circRNAs in four RCC and matched normal tissues using arrays that only contain probes for circRNAs. Despite this progress, the mechanistic functions of circRNAs in RCC have not been deeply explored.

Here, we identified 387 differentially expressed circRNAs in metastatic RCC by using circ-microarray. In this dataset, a significantly upregulated circRNA, termed circPRRC2A, is identified for the first time to be critical for mRCC. circPRRC2A is generated from the PRRC2A gene at 6p21.33 amplicon. circPRRC2A is correlated with lymph node metastasis and advanced TNM stage in RCC patients. Subsequently, a biotin- coupled miRNA pulldown experiment determined that circPRRC2A may sponge both miR-514a-5p and miR-6776-5p, thereby increasing TRPM3 and inducing epithelial-mesenchymal transition (EMT) and tumor angiogenesis in RCC. Our findings demonstrate that circPRRC2A exhibits oncogenic properties, is a candidate to aid in the diagnosis of RCC, may be a prognostic indicator, and is a potential therapeutic target for RCC.

## Materials and Methods

### RCC patient samples

All primary RCC tumor tissues with matched normal-adjacent renal tissues were derived from 118 patients with RCC who had received operative treatment at Shanghai Tenth People's Hospital of Tongji University (Shanghai), Beijing Chao-Yang Hospital of Capital Medical University (Beijing), and Tongren Hospital of Shanghai JiaoTong University School of Medicine (Shanghai) from 2006 to 2016 (Table S1). All RCC cases were confirmed by a senior pathologist, and staged based upon the American Joint Committee on Cancer (AJCC) 8th edition (2019) 22. Human tissue collection was conducted in accordance with the International Ethical Guidelines for Biomedical. This study was approved by the Ethics Committee of above-mentioned Hospitals and was conducted in concordance with the provisions of the Ethics Committee of Tongji University (*No.*SHSY-IEC-BG/02.04/04.0-81602469). Written informed consent was obtained from the patients before the study began.

### Total RNA isolation, qRT-PCR and RNA-seq analysis

Total RNA from whole-cell lysates, nuclear, or cytoplasmic fractions were isolated in triplicate using TRIzol reagent (Invitrogen) for cultured cells or using the GeneJET RNA Purification Kit (Invitrogen) for fresh-frozen tissues. Real-time quantitative PCR was conducted using the 2×SYBR Green qPCR Master Mix (Tiangen, China). Differential expression of circRNAs and linear RNAs was calculated using the ^ΔΔCt^ method, and normalized relative to GAPDH and small nuclear U6 levels. The primers are presented in Table S3. The microarray data are deposited in the NCBI Gene Expression Omnibus (GEO) datasets under the accession number GSE137836.

### Statistical analysis

All data were processed by SPSS 19.0 software (SPSS, Inc., Chicago, USA). Correlations were measured by Pearson correlation analysis. Differences between groups were determined by a paired twotailed *t* test. One-way ANOVA or the nonparametric Kruskal-Wallis test was used to evaluate the relationship between circPRRC2A levels and other features. The Kaplan-Meier method was used to calculate survival curves, and differences were determined by a log-rank test. The Cox proportional hazards model was utilized to determine the independent factors, that were based on the variables indicated by a univariate analysis. Statistical significance was indicated by *P* values less than 0.05 as: ^*^P <0.05, ^**^P <0.01.

## Results

### Expression profiles and integrated screening of circRNAs in human RCC tissues

**Figure 1:**
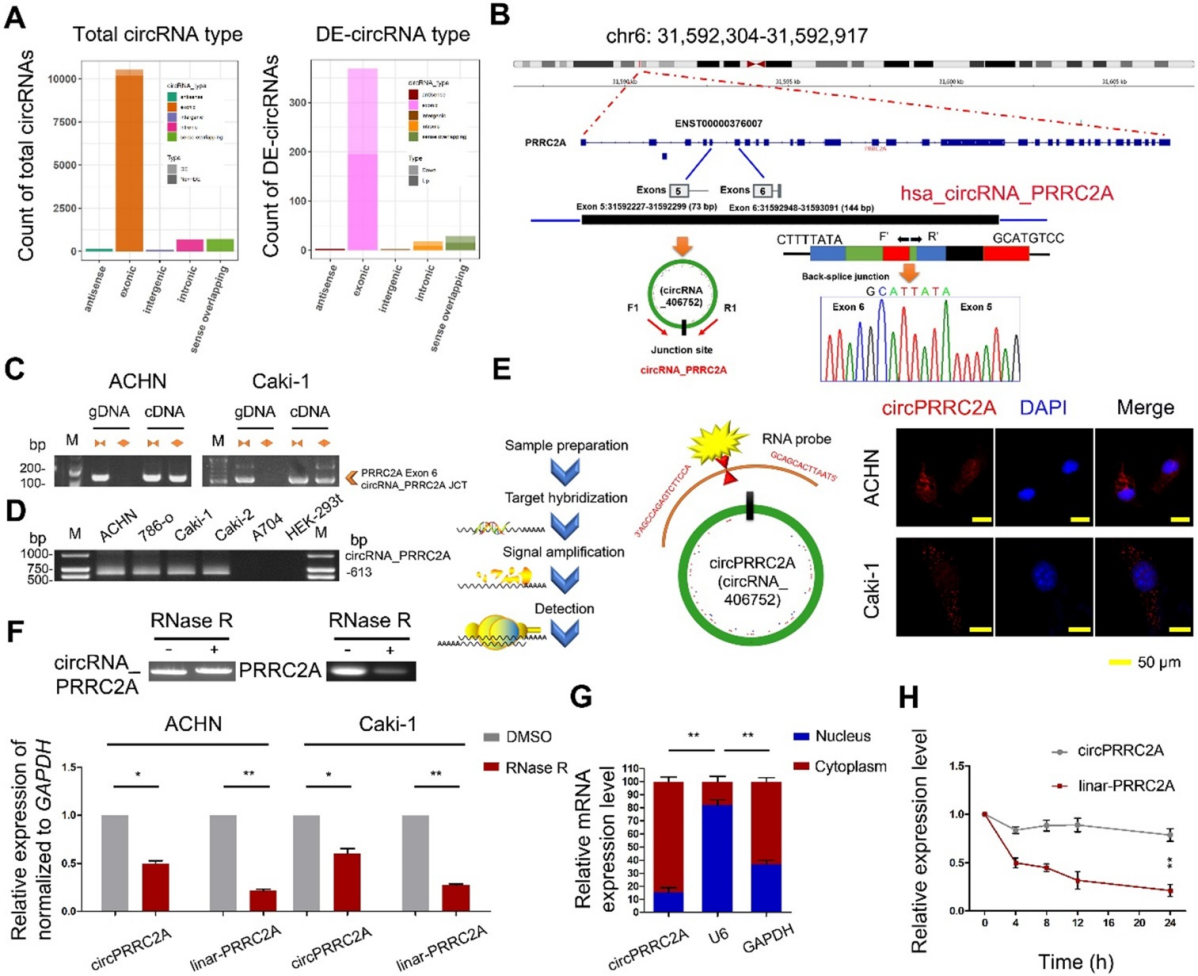
circRNA expression profile in mRCC and characterization of circPRRC2A. **A** Genomic origin of differentially expressed circRNAs. DE (Differentially expressed circRNAs). **B** The schematic diagram of genomic location and splicing pattern of circPRRC2A. The splice junction was verified by Sanger sequencing. circPRRC2A is back-spliced by exons 5 and 6 of PRRC2A, PCR primers used to specifically detect circPRRC2A by qRT-PCR are indicated by red arrows. **C** qRT-PCR products with divergent primers showing circularization of circPRRC2A. The divergent primers detected circular RNAs in cDNA but not in gDNA. gDNA, genomic DNA; cDNA, complementary DNA. **D** The existence of circPRRC2A was validated in RCC cells. **E** Representative FISH images of circPRRC2A, shown localization of circPRRC2A in the cytoplasm of RCC cells. Nuclei were stained with DAPI. Scale bar, 50 μm. **F** qRT-PCR analysis of circPRRC2A and PRRC2A mRNA after treatment with or without RNase R in RCC cells. circPRRC2A was resistant to RNase R treatment. **G** Cytoplasmic and nuclear mRNA fractionation experiment showed that circPRRC2A is mainly located in the cytoplasm. The amount of circPRRC2A was normalized to the value measured in the cytoplasm, n = 3. **H** The levels of circPRRC2A and linear-PRRC2A expression in ACHN cells treated with Actinomycin D (2.5 μg/ml) at the indicated time points were detected by qRT-PCR. All data are presented as means ± SD. Student's t test was used. *P < 0.05, **P < 0.01.

To identify circRNAs that are differentially expressed (DE) in RCC, we initially evaluated circRNA transcripts using microarray analysis on ribosomal RNA-depleted, RNase R (digests linear RNAs leaving only circRNAs intact) treated RNA from three paired metastatic human RCCs (mRCCs) and matched primary RCC tissues. A total of 418 dysregulated circRNAs were identified (219 circRNAs were upregulated and 199 circRNAs were downregulated; *P* < 0.05, and fold change > 2.0). Out of the total 418 DE circRNAs in mRCCs compared with matched RCC tissues, the majority were derived from exons (Figure 1A). To investigate the putative pathways that circRNAs are implicated in within mRCCs, we performed a co-expression network analysis between dysregulated circRNAs and mRNA in tumor metastasis. The effects of the DE circRNAs on various biological functional gene sets were analyzed by the Gene set enrichment analysis (GSEA). In the GSEA analysis of KEGG and GO enrichment biological processes pertaining to positive regulation of renal cell carcinoma, cell differentiation, and cell adhesion, were significantly enriched. To validate the microarrays results, we selected the top 10 upregulated and downregulated circRNAs. Nine upregulated and nine downregulated circRNAs were successfully validated in RCC tissues using divergent primers in quantitative reverse transcription PCR (qRT-PCR) that specifically only amplify circRNAs followed by Sanger sequencing. Notably, circPRRC2A was significantly upregulated according to the probe signal calculation of the microarray data. The increased expression of circPRRC2A in mRCCs was confirmed in a total of 118 paired RCC samples by qRT-PCR. circPRRC2A was significantly higher in 80.5% (95 of 118) of RCC tissues (Table S2).

### Characterization of circPRRC2A in mRCC cells

We chose circPRRC2A for further study for the following reasons: (a) circPRRC2A is among the most abundant circRNAs that were DE in our microarray data and its absolute quantification in mRCCs. (b) GSEA analysis suggested that circPRRC2A could regulate tumor cell adhesion and the VEGF signaling pathway. VEGF is of crucial importance in the initial events of metastatic dissemination of tumor cells by giving them greater motility and invasiveness. We next determined that circPRRC2A is generated from exon 5 to 6 of the PRRC2A gene. The back-spliced junction of circPRRC2A was amplified with divergent PCR primers and validated by Sanger sequencing (Figure 1B).

circPRRC2A was further characterized using either divergent primers that amplify only circular transcripts or convergent primers that detect linear RNA molecules on either cDNA or gDNA. While circPRRC2A was detected with divergent primers in cDNA but not gDNA, convergent primers amplified both cDNA and gDNA (Figure 1C). Furthermore, we determined that the endogenous expression level of circPRRC2A is elevated in RCC cell lines compared with normal human renal cell lines (Figure 1D). In addition, mRNA fractionation and Fluorescence In Situ Hybridization (FISH) for circPRRC2A demonstrated a predominantly cytoplasmic localization of circPRRC2A (Figure 1E and 1G). Expectedly, linear PRRC2A RNA was degraded by RNase R, while circPRRC2A was resistant to RNase R degradation (Figure 1F). Analysis of circPRRC2A and PRRC2A RNA stability in ACHN cells treated with Actinomycin D, an inhibitor of transcription, revealed that the half-life of circPRRC2A transcript exceeded 24hrs, and was far more stable than PRRC2A (Figure 1H).

### Correlation between circPRRC2A and clinical characteristics of RCCs, and the expression of circPRRC2A in mRCCs can be regulated by DHX9

**Figure 2:**
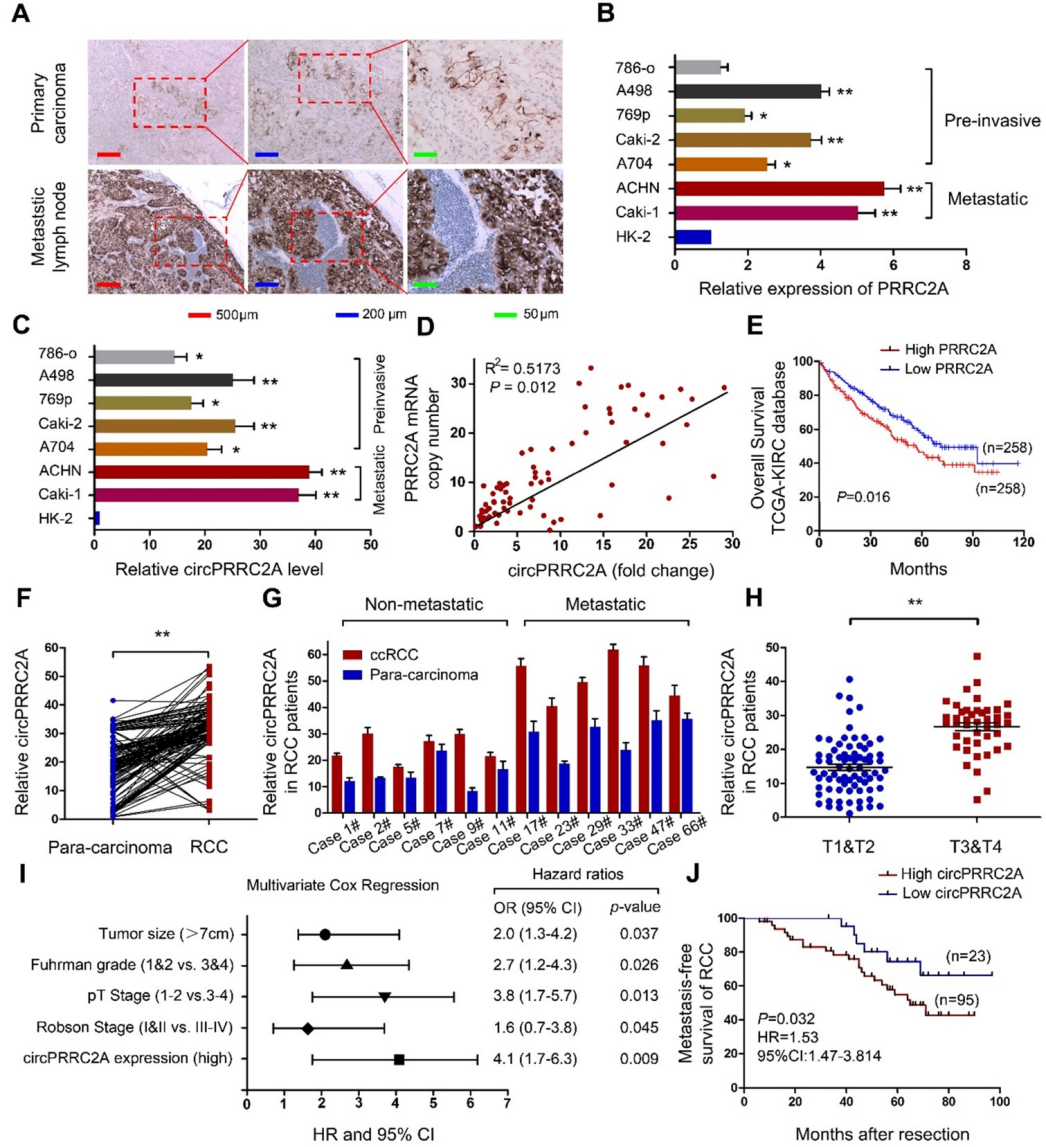
circPRRC2A is frequently upregulated due to gene amplification of PRRC2A in clinical RCC specimens and cell lines, and correlates with poor patient prognosis. **A** IHC assay was performed to detect the expression of PRRC2A in mRCC tissues. PRRC2A was upregulated in metastatic lymph nodes compared with primary tissues, n = 21.** B**-**C** Expressions of PRRC2A and circPRRC2A were measured in RCC cell lines using qRT-PCR. **D** Positive correlation between PRRC2A genomic DNA content level and circPRRC2A expression level in mRCC tissues. **E** Kaplan-Meier's survival curve indicated the high PRRC2A expression is correlated with lower RCC survival rates. **F** The levels of circPRRC2A expression were detected in RCC and para-carcinoma (n = 118). **G** circPRRC2A expressions was evaluated using qRT-PCR in non-metastatic RCC patients and metastatic patients tissue. **H** Determination of circPRRC2A expression in early stage RCC tumors (n=67) and advanced RCC tumor (n=51) by qRT-PCR. **I** Multivariate Cox regression of hazard ratios for mRCC OS. OS, overall survival. **J** Kaplan-Meier's survival curves showed the correlations between circPRRC2A expression and MFS. MFS, metastasis-free survival. Log-rank test was used. Data indicate mean ± SD, n = 3; *P < 0.05, **P < 0.01.

It has been reported that the amplification of host genes is related to the expression level of circRNAs 23,24. To investigate the correlation between PRRC2A gene amplification and circPRRC2A expression, we first measured the expression level of PRRC2A in RCCs and mRCCs by IHC. The results showed that the expression of PRRC2A in metastatic lymph node was significantly greater than in primary carcinoma (Figure 2A). Then we evaluated the copy number of PRRC2A and circPRRC2A expression in RCC cell lines. We observed amplification of the PRRC2A gene in ACHN, Caki-1, A498 and Caki-2 cells relative to the internal control HK-2 cell (Figure 2B). Interestingly, circPRRC2A expression was significantly higher in these four cell lines compared with other RCC cell lines (Figure 2C). Importantly, the PRRC2A copy number was amplified in metastatic tumor tissues compared with paired primary cancer tissues and positively correlated with circPRRC2A expression (R^2^ = 0.5173, *P* = 0.012; Figure 2D). Kaplan-Meier survival analysis confirmed that patients with higher PRRC2A levels had a worse overall survival rate than those with lower PRRC2A levels from The Cancer Genome Atlas (TCGA) database (*P* = 0.016) (Figure 2E). Then, the expression of circPRRC2A was measured in 69 primary RCCs and para-carcinoma tissues by qRT-PCR. circPRRC2A was highly elevated in RCC tissues (*P* < 0.01; Figure 2F). Next, we investigated the association between circPRRC2A and clinical and pathologic parameters. Tumors that are metastatic (Figure 2G) or are higher in TNM stage (T3-T4 vs T1-T2; *P* < 0.01, Figure 2H) displayed higher expression of circPRRC2A. Furthermore, multivariate analyses indicated that the circPRRC2A expression level was an independent risk factor for overall survival, together with RCC tumor size, Fuhrman grade, and pT stage. These analyses also indicated that circPRRC2A expression, together with tumor size, Fuhrman grade, and Robson stage, were independent risk factors for metastatic free survival (Table S2, Figure 2I, 2J).

We investigated why circPRRC2A was significantly upregulated in mRCCs. Since circRNAs are derived from pre-mRNAs and circRNAs can be regulated by RNA-binding proteins post- transcriptionally, we determined the expression of circPRRC2A in RCC cell lines after individually depleting all three known human circRNA-binding proteins (QKI, ADAR1 and DHX9) that have been shown to regulate circRNA biogenesis 25-28. We found that DHX9 was down-regulated in RCC tissues comparatively but ADAR1 and QKI had no significant difference (Figure S2A, Figure S3A). DHX9 is an abundant intranuclear RNA-binding gene that could bind to the inverted repeat Alu element of RNA to prevent the production of circRNAs 19,29. With the increase of RCC stage and metastatic status, the expression level of DHX9 is further decreased (Figure S2B, S2C). Importantly, low expression of DHX9 was associated with worse overall survival (Figure S2D). DHX9 was obviously downregulated in our clinical mRCC (Figure S2E-S2H). Notably, after knocking down DHX9 but not ADAR1 or QKI, circPRRC2A was significantly upregulated (Figure S2I, S2J and Figure S3B-S3E). Overexpression of a wild-type DHX9 transgene could reverse this effect (Figure S2K, S2L). Taken together these results indicate that the upregulation of circPRRC2A was, at least in part, caused by the downregulation of DHX9 in mRCCs.

### circPRRC2A promotes angiogenesis and metastasis *in vitro* and *in vivo*

**Figure 3:**
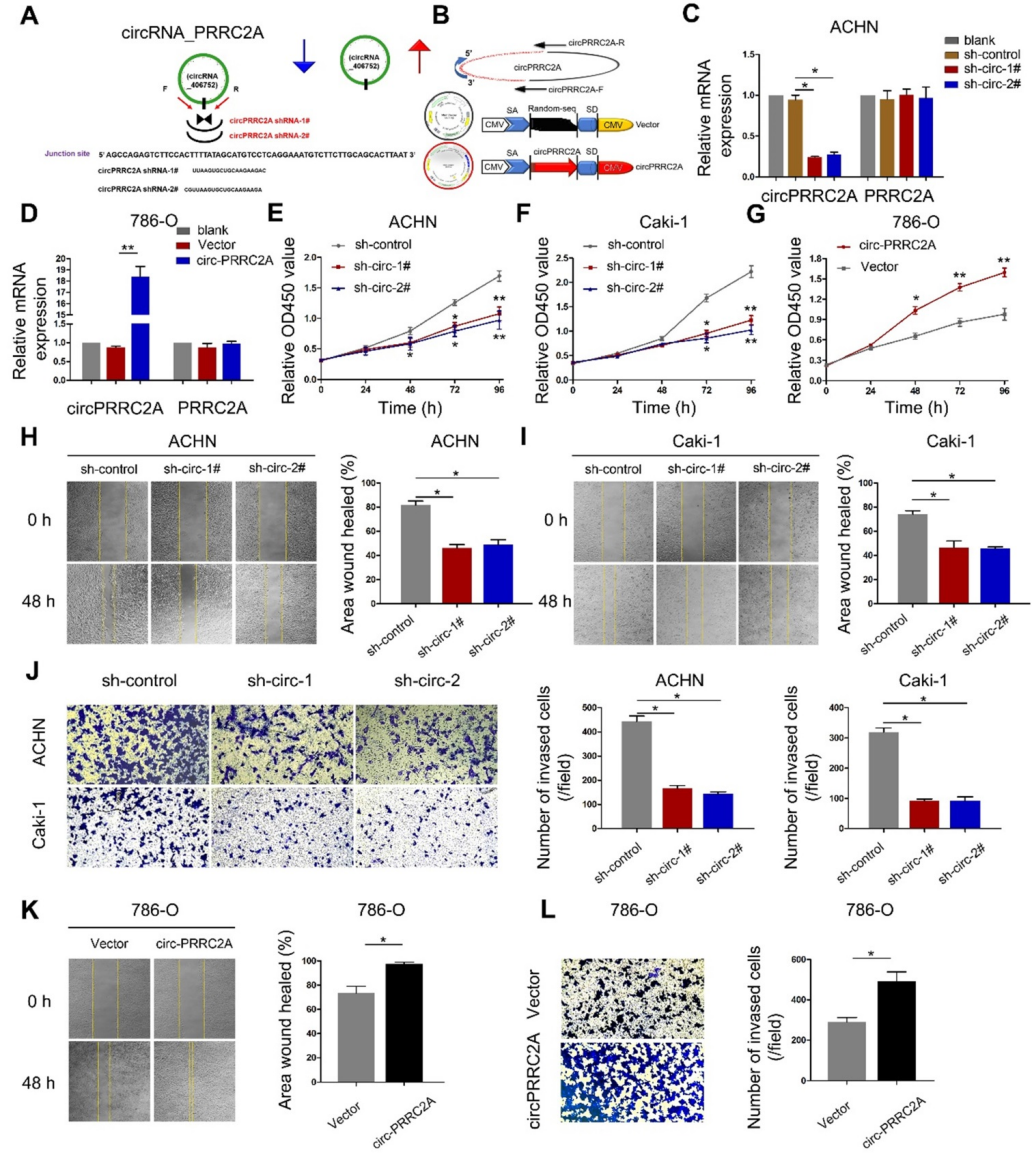
circPRRC2A promotes the migration and invasion of renal cancer cells *in vitro*. **A** Schematic illustration showed two targeted shRNAs and circPRRC2A expression vectors **B** circPRRC2A-shRNA targets the back-splice junction of circPRRC2A. **C** qRT-PCR for PRRC2A and circPRRC2A mRNA in ACHN cells treatedwith two shRNAs as described above. **D** qRT-PCR for PRRC2A and circPRRC2A mRNA in 786-o cells transfected with control vector or circPRRC2A over-expression plasmid. **E**-**G** Growth curves of RCC cells were measured after transfection with indicated vectors by Cell Counting Kit-8 assays (CCK-8). **H**-**I** Wound-healing assays showed that knockdown of circPRRC2A inhibited the migration ACHN and Caki-1 RCC cells. **J** Transwell assays showed that knockdown of circPRRC2A inhibited the metastasis of ACHN and Caki-1 cells. Wound-healing **K** and transwell assays **L** showed that overexpression of circPRRC2A promoted the invasion ability of the 786-o cells. Data indicate mean ± SD, n = 3; *P < 0.05, **P < 0.01.

To study the role of circPRRC2A in RCC progression, we first constructed a circ-shRNA, covering the back-spliced region of circPRRC2A (Figure 3A). The results showed that shRNA knockdown of circPRRC2A resulted in a marked decrease in its RNA level. Also, to ectopically overexpress circPRRC2A, exon 5 and 6 of PRRC2A were cloned into the lentiviral vector (Figure 3B, Figure S1). We further investigated effects on the 5' neighboring genes of PRRC2A and found that circPRRC2A depletion or forced expression had no effect on neighboring genes (Figure 3C, 3D). However, silencing of circPRRC2A could significantly inhibit the proliferation and migration of ACHN and Caki-1 cells (Figure 3E, 3F and 3H, 3I). Matrigel invasion assay revealed that silencing of circPRRC2A significantly suppressed cell invasion capacity (Figure 3J). Conversely, stably overexpressing circPRRC2A in RCC 786-o cells markedly increased cell proliferation, migration and invasion (Figure 3G and 3K, 3L).

**Figure 4:**
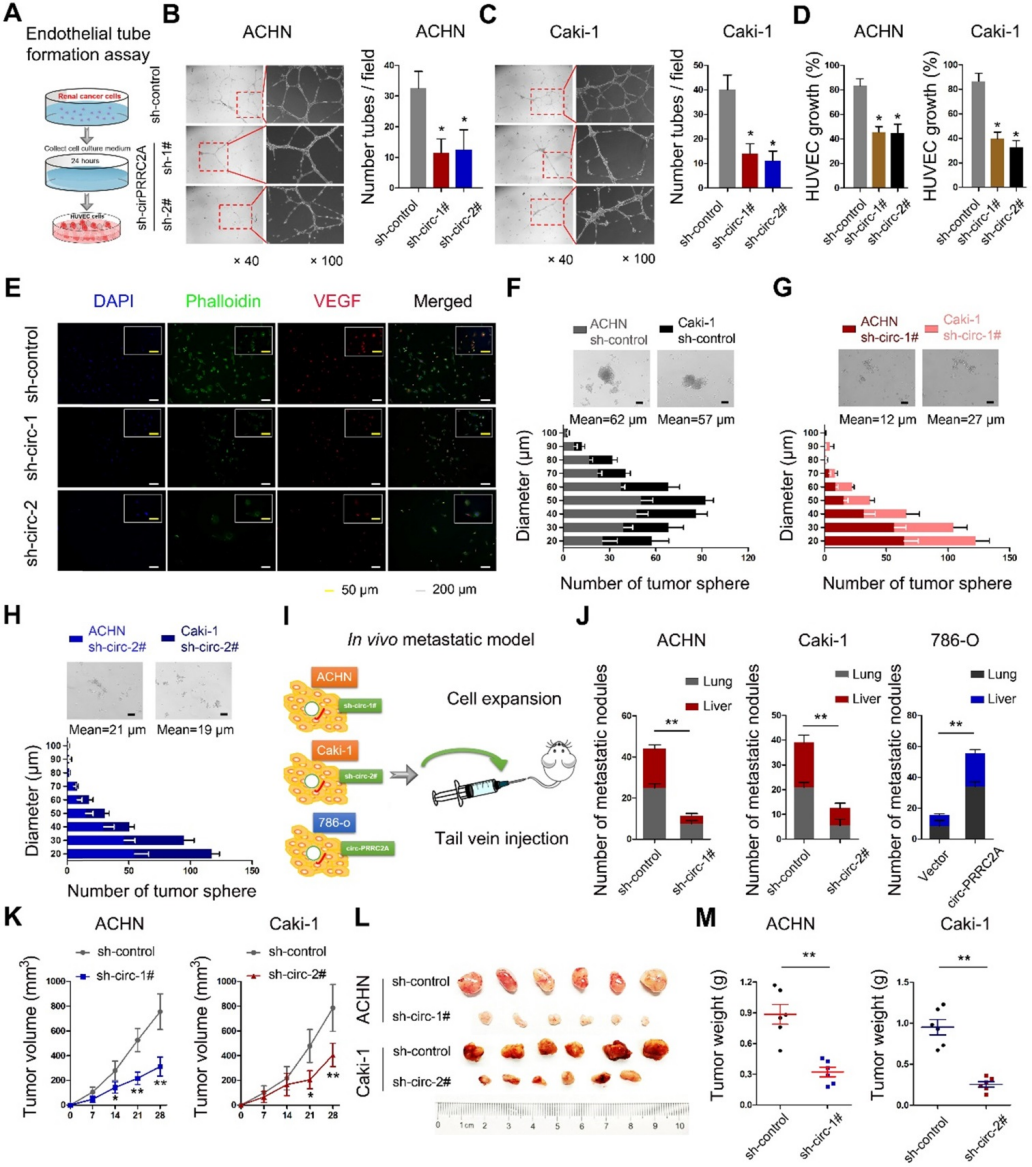
circPRRC2A exerts oncogenic effects of renal cancer cells *in vitro* and* in vivo*. **A** Graphic illustration of tube formation assay. **B**-**C** Tube formation assay in HUVECs cultured for 24 h with medium collected from RCC cells with sh-control and sh-circPRRC2A. Tubules were imaged and quantification of tube formation and cell growth was performed. **D** in HUVECs cultured for 24 h with medium from RCC cells with sh-control and sh-circPRRC2A. **E** Immunofluorescence for VEGFA in HUVECs cultured for 24 h in medium from ACHN cells were performed. **F**-**H** Effect of circPRRC2A on the growth of RCC cells in the 3-D tumor sphere formation and tumor sphere growth assay. The size profile of RCC tumor spheres were observed after 7 days. The number and diameter of tumor spheres were measured after sh-circPRRC2A transfection and presented as the size profile of tumor sphere. The mean diameters of tumor spheres with sh-circ-1 and sh-circ-2 were significantly smaller than the sh-control (*P* < 0.01, respectively). **I** Graphic illustration of the *in vivo* metastasis mice model study. **J** Stably transfected ACHN/Caki-1 cells with sh-circRNA1# or 2# and 786-o with overexpression of circPRRC2A with their respective control lentivirus were injected into the caudal vein of BALB/c nude mice for 8 weeks. The number of metastatic nodules formed in the lungs and livers of BALB/c nude mice is summarized for each group tested. The subcutaneous xenotransplanted tumor model analyses of tumor growth (**K**, two-way ANOVA followed by Bonferroni's test), tumor specimens **L**, or weight (**M**, independent-samples *t* test) in nude mice that were subcutaneously injected with ACHN/Caki-1 cells harboring sh-control or sh-circRNA. n = 6 mice per group. Data indicate mean ± SD of three experiments. *P < 0.05, **P < 0.01.

To identify circPRRC2A-regulated genes that are involved in tumor angiogenesis, we examined the expression of vascular endothelial growth factor (VEGF) and the effects of tube formation after knocking down circPRRC2A (Figure 4A). When human umbilical vein endothelial cells (HUVECs) were cultured in the media conditioned by cells with circPRRC2A shRNA knockdown, both tube formation and growth were significantly impaired (Figure 4B, 4C). Further, loss of circPRRC2A dramatically reduced the proliferative capacity of HUVECs (Figure 4D). Immunofluorescence assays also confirmed that circPRRC2A knockdown caused changes in HUVECs morphology and the expression level of VEGF (Figure 4E). Next, the tumorsphere formation assay showed that loss of circPRRC2A significantly inhibited the formation of tumorspheres (Figure 4F-4H). The role of circPRRC2A in tumor metastasis was evaluated *in vivo* using BALB/c nude mice injected intravenously in the tail vein with RCC cells expressing either sh-circPRRC2A or overexpression of circPRRC2A (Figure 4I). 40 days after cell injection, tumor biopsies showed that the mice injected with cells with sh-circPRRC2A had less metastatic nodules in the lung and liver than mice injected with control cells. Meanwhile, mice injected with cells with stable overexpression of circPRRC2A had markedly increased tumor nodules in their lungs and livers (Figure 4J). Subsequently, to explore the effects of circPRRC2A on tumor growth, we performed subcutaneous tumor formation in nude mice. The result showed that the growth of tumors from sh-circPRRC2A was significantly inhibited (Figure 4K-4M, Figure S4).

### circPRRC2A promotes the angiogenesis and metastasis through the miR-514a-5p/ miR-6776-5p-TRPM3 pathway in RCC

**Figure 5:**
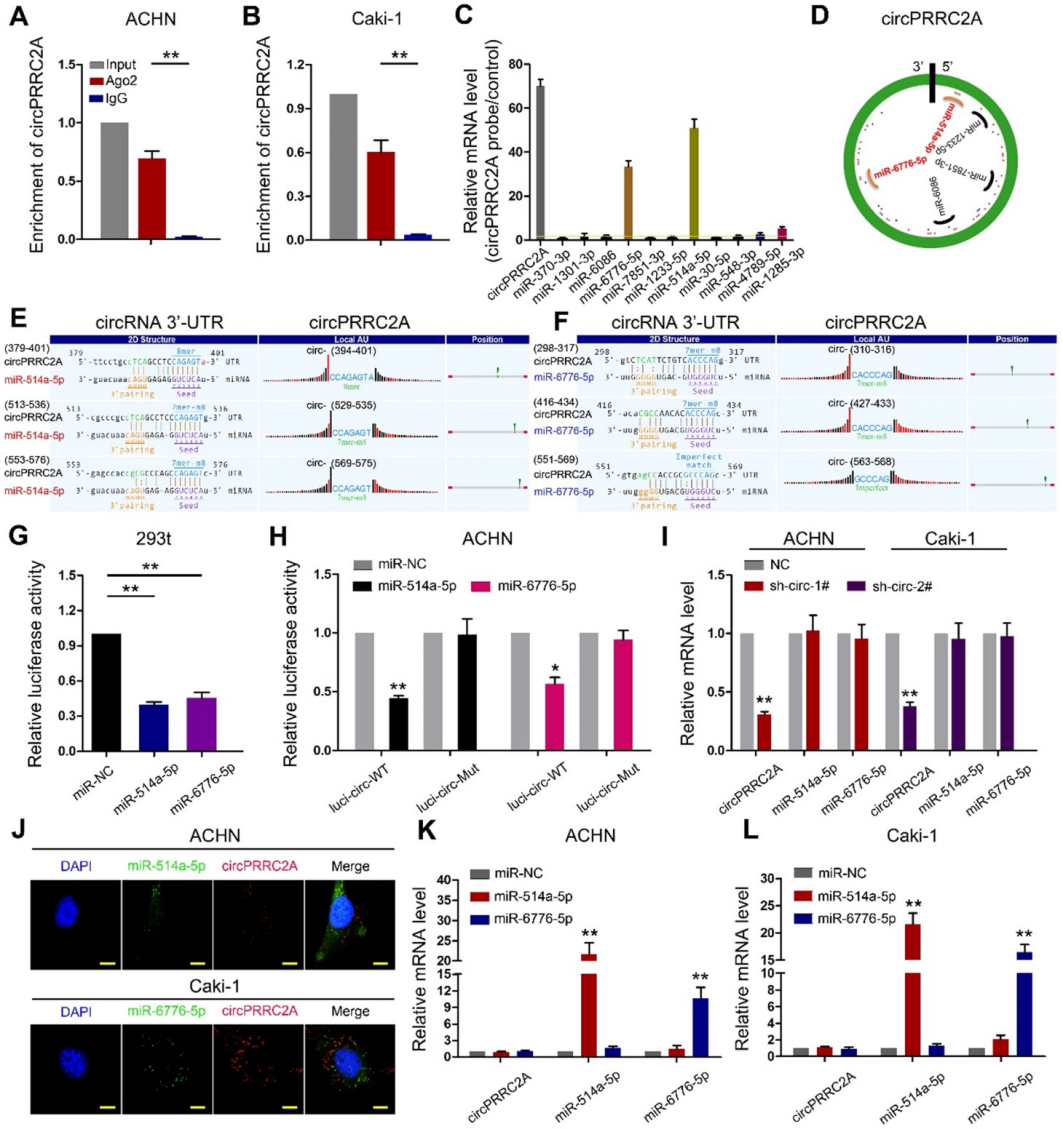
circPRRC2A function as a sponge for miR-514a-5p and miR-6776-5p in RCC cells. RNA RIP experiments were performed using an antibody against Ago2 onextracts from ACHN **A** and Caki-1 **B** cells. **C** RNA RIP experiments were performed in ACHN cells using the circPRRC2A probe or a control probe. The enrichment of circPRRC2A and potential target microRNAs were detected by qRT-PCR and normalized relative to the control (one-way analysis of variance, Dunnett's test). **D** Schematic drawing showing the potential miRNAs that might bind circPRRC2A. **E**-**F** Schematic of the predicted miR-514a-5p (L) and miR-6776-5p (R) sites in the circPRRC2A. **G** Luciferase reporter activity of Luc-circPRRC2A in 293t cells after transfection with miR-514a-5p and miR-6776-5p. **H** Luciferase reporter activity of Luc-circPRRC2A-WT (wildtype) or Luc-circPRRC2A-Mut (mutant) in ACHN cells after transfection with miR-514a-5p and miR-6776-5p. **I** Silencing of circPRRC2A did not affect the expression level of miR-514a-5p or miR-6776-5p.** J** Co-localization between miR-514a-5p, miR-6776-5p and circPRRC2A was detected by FISH assay in ACHN and Caki-1 cells. Nuclei were stained with DAPI. **K**-**L** miR-514a-5p and miR-6776-5p did not affect the expression level of circPRRC2A, Student *t* test. Data indicate mean ± SD of three experiments. *P < 0.05, **P < 0.01.

Since circRNAs are known to sponge miRNAs and circPRRC2A is stable and localized in the cytoplasm, we first performed RNA immunoprecipitation (RIP) using an argonaute 2 (AGO2) antibody in RCC cells. circPRRC2A was significantly enriched by the AGO2 antibody (Figure 5A, 5B). Therefore, we constructed a circPRRC2A-miRNAs network by miRanda prediction (Table S4). We then purified the circPRRC2A-associated RNAs, using circPRRC2A specific probes, and analyzed the 12 potential candidate miRNAs. We detected an enrichment of miR-514a-5p and miR-6776-5p binding to circPRRC2A relative to the controls, while the other miRNAs demonstrated no enrichment in ACHN cells (Figure 5C, 5D). We also generated constructs wherein the target sites for miR-514a-5p or miR-6776-5p were mutated (Figure 5E, 5F). Compared with the control, miR-514a-5p or miR-6776-5p reduced the luciferase reporter activity by at least 60% (Figure 5G). Additionally, the luciferase activities of the circPRRC2A wild type reporter were significantly reduced when transfected with miR-514a-5p or miR-6776-5p mimics compared with mutated luciferase reporters (Figure 5H). Moreover, a double FISH assay demonstrated co-localization of circPRRC2A and miR-514a-5p/miR-6776-5p (Figure 5J). Interestingly, silencing of circPRRC2A did not affect the expression level of miR-514a-5p or miR-6776-5p, and transfection of miR-514a-5p or miR-6776-5p mimics did not affect the expression of circPRRC2A (Figure 5I and 5K, 5L). Taken together, our data provide evidence that circPRRC2A may function as a sponge for miR-514a-5p and miR-6776-5p.

**Figure 6:**
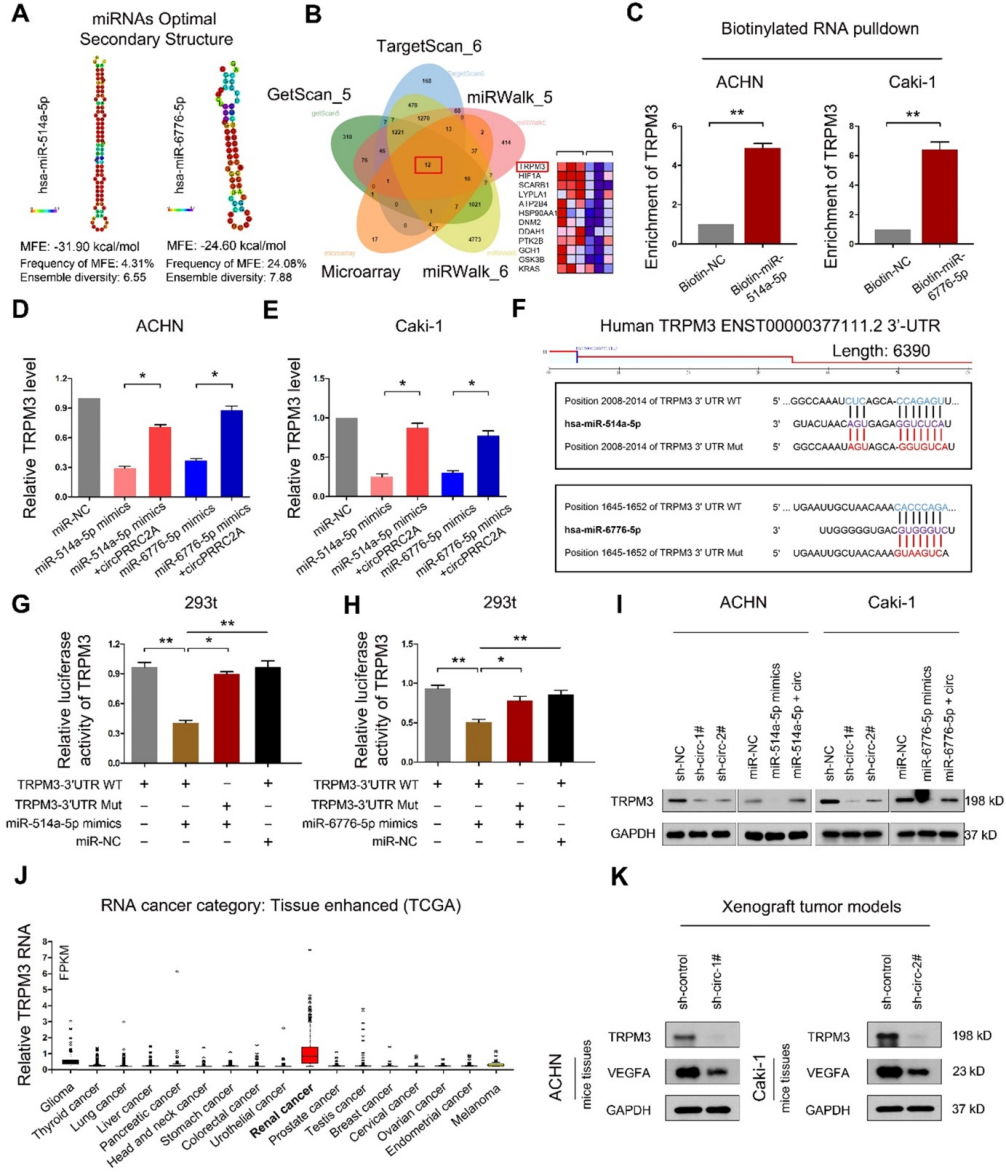
circPRRC2A serves as a sponge for miR-514a-5p and miR-6776-5p to increase TRPM3 in RCC. **A** Secondary structure and sequence conservation predictions of miR-514a-5p and miR-6776-5p. **B** Venn diagram showing the mutual putative target genes of miR-514a-5p and miR-6776-5p. TRPM3 is a mutual putative target gene of miR-514a-5p and miR-6776-5p, Arraystar Human circRNA Array analysis also showing that TRPM3 is differentially expressed in mRCC (middle, heat map). **C** Biotinylated RNA pulldown assay demonstrates that the miR-514a-5p or miR-6776-5p-captured fractions distinctly enrich TRPM3. **D**-**E** miR-514a-5p or miR-6776-5p mimics could significantly suppress the expression level of TRPM3, and this suppression was reversed after over-expression of circPRRC2A. **F** Schematic of TRPM3 wild-type (WT) and mutant (Mut) luciferase reporter vectors. **G**-**H** Relative luciferase activities were analyzed in 293t cells co-transfected with miR-514a-5p mimics, miR-6776-5p mimics, or miR-NC and WT or Mut luciferase reporter vectors. **I** Western blot analysis showed sh-circPRRC2A or miR-514a-5p/miR-6776-5p mimics could significantly suppresses the expression level of TRPM3, and the suppression was reversed after over-expression of circPRRC2A. **J** Relative expression levels of TRPM3 in tumor patients derived from TCGA database. **K** Western blot analysis was used to detect the TRPM3 and VEGFA protein levels in Xenograft tumor tissues transfected with the sh-circPRRC2A compared with the expression in their corresponding controls. Data indicate mean ± SD. *P < 0.05, **P < 0.01.

RNAfold software prediction results display that both miR-514a-5p and miR-6776-5p have mature and stable secondary structures (Figure 6A). We used the TargetScan and miRWalk prediction programs to confirm the downstream target genes of miR-514a-5p and miR-6776-5p (Table S5-S6). A Venn diagram using these 5 gene sets identified 12 candidate target mRNAs (Figure 6B). In this analysis, TRPM3 displayed the most significant difference. To determine whether TRPM3 is a direct target of miR- 514a-5p and miR-6776-5p, we employed a miRNA- biotin pull-down experiment and demonstrated that both miR-514a-5p and miR-6776-5p could significantly enrich TRPM3 mRNA (Figure 6C). We then observed that exogenous miR-514a-5p or miR-6776-5p could significantly suppress TRPM3 expression and that this suppression was reversed upon overexpression of circPRRC2A (Figure 6D, 6E). We build wild-type and mutant 3'-UTRs of TRPM3 mRNA and found that miR-514a-5p or miR-6776-5p mimics efficiently suppressed luciferase activity of the wild-type reporter but not the mutant (Figure 6F-6H). These results indicated that both miR-514a-5p and miR-6776-5p bind to the 3'-UTR of TRPM3. Upon transfection of either miR-514a-5p/miR-6776-5p mimics or sh-circPRRC2A, the protein level of TRPM3 was also significantly decreased, and that the inhibiting effect was reversed after overexpression of circPRRC2A (Figure 6I). TRPM3 mRNA levels are significantly upregulated in RCC compared to other tumors from the TCGA database (Figure 6J). Moreover, we examined the expression of TRPM3 in tumor tissues of nude mice. The results showed that silencing of circPRRC2A inhibited the protein expression of TRPM3 and VEGFA (Figure 6K). TRPM3 is a critical oncogene that is upregulated in metastatic RCC and could promote the metastasis of RCC and predict poor survival 30,31. Functionally, colony formation, wound healing and transwell assays showed that miR-514a-5p/miR-6776-5p could inhibit the proliferation, invasion and metastasis of RCC cells and that this inhibition could be abrogated by forced expression of circPRRC2A (Figure S5). These findings reveal that circPRRC2A promotes the invasion and metastasis of RCC, through the miR-514a-5p/miR-6776-5p-TRPM3 pathway.

### circPRRC2A regulates the TRPM3/SNAIL/Vimentin pathway and promotes EMT progression in RCC cells

**Figure 7:**
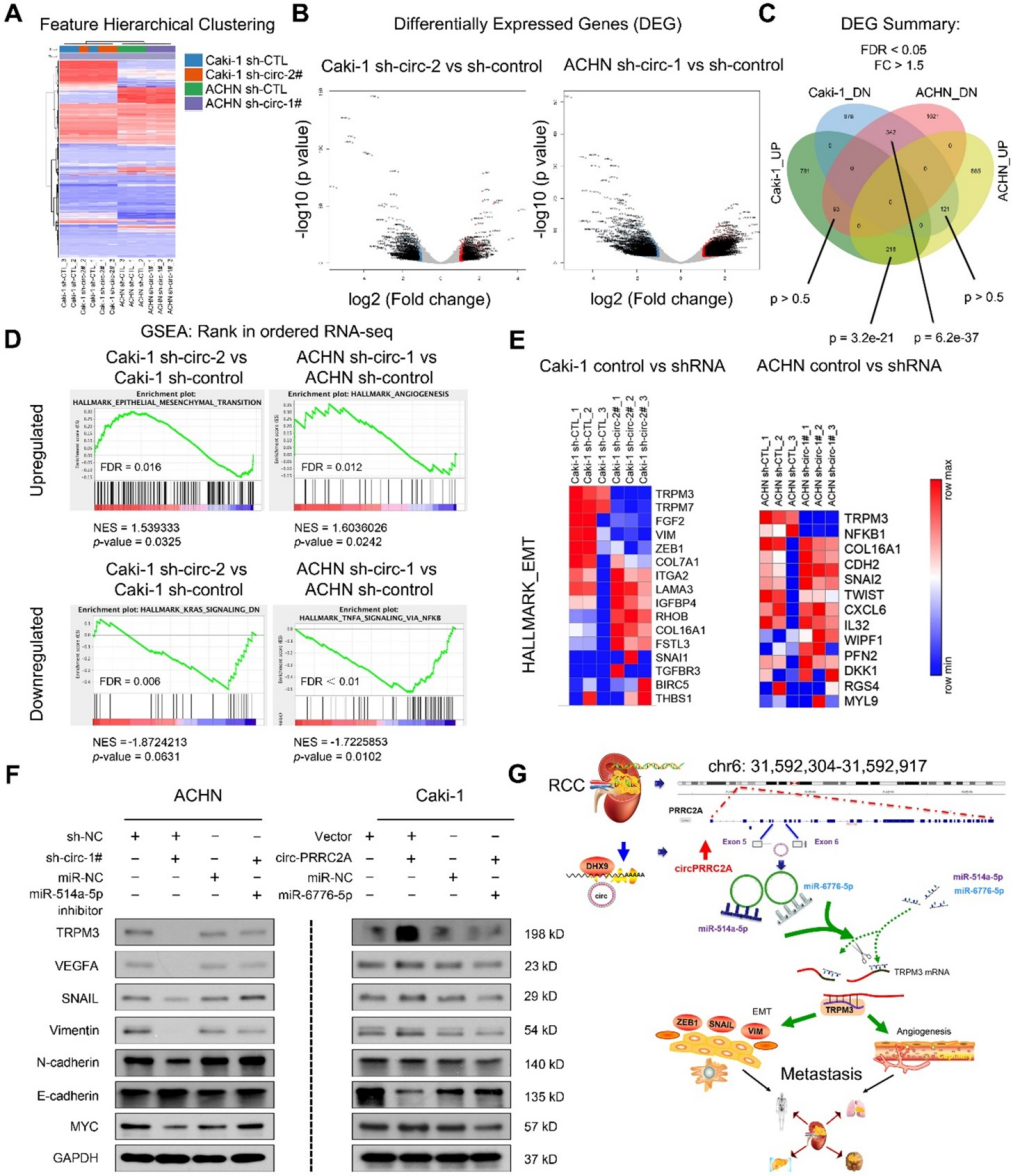
RNA-sequencing showed circPRRC2A could promote the angiogenesis and metastasis of RCC through the miR-514a-5p/miR-6776-5p/TRPM3 pathway. **A** Expression profile of circPRRC2A-regulated oncogenes using the RNA-seq in ACHN/Caki-1/sh-CTL and ACHN/Caki-1/sh-circRNA cells. **B**-**C** A volcano map shows differentially expressed genes (DEG). There is a significant overlap of circPRRC2A associated gene expression changes in ACHN and Caki-1 cell lines. **D** RNA-seq analyses were performed in triplicate on ACHN and Caki-1 cells. The datasets were analyzed by GSEA, using the Hallmark gene signature collection. GSEA indicating significant correlations between circPRRC2A expression and tumor metastasis-related gene signatures. **E** RNA-seq analyses of circPRRC2A knockdown in RCC cell lines, significantly enriched Hallmark_EMT pathways in both cell types. **F** The expressions of TRPM3, VEGFA and EMT-related makers and stemness-related proteins were determined using western blot. Proteins were isolated from cells transfected as indicated. Data indicate mean ± SD, n = 3. **G** Molecular mechanism of circPRRC2A. PRRC2A gene amplification leads to increased expression of circPRRC2A in mRCC tissues. circPRRC2A sponges miR-514a-5p and miR-6776-5p, thereby regulating angiogenesis and EMT processes via increasing TRPM3 activity in RCC cells' invasion and metastasis. *P < 0.05, **P < 0.01. GSEA, gene set enrichment analysis, EMT, Epithelial-Mesenchymal Transition.

To extend the findings that circPRRC2A promote RCC metastasis, RNA-seq analysis was performed on cells with/without circPRRC2A silencing (Figure 7A, 7B). A total of 216 upregulated differential expressed genes (DEGs) and 342 downregulated DEGs were identified (Figure 7C). Analysis of these data using the Hallmark Molecular Signature Database and Kyoto Encyclopedia of Genes and Genomes (KEGG) showed that circPRRC2A plays a significant role in the regulation of EMT and cell adhesion. Importantly, GSEA analysis of RNA-seq data demonstrated that knockdown circPRRC2A correlates significantly with downregulation of EMT target genes, also confirmed that circPRRC2A is positively correlated with tumor angiogenesis and negatively correlated with the KRAS and TNF-α pathways (Figure 7D, 7E). To confirm the association of circPRRC2A with EMT pathway-related genes, western blot analysis showed that in the knockdown of circPRRC2A in ACHN cells, the expression of the epithelial marker, E-cadherin was increased, whereas the expression of the mesenchymal markers, TRPM3, Snail, Vimentin, MYC, VEGFA and N-cadherin, were decreased (Figure 7F, left). Conversely, overexpression of circPRRC2A led to opposite results (Figure 7F, right). In addition, we analyzed the correlation between TRPM3 with these EMT related genes from TCGA database 32,33. These data indicate that circPRRC2A promotes RCC metastasis through the miRNAs- TRPM3/SNAIL/Vimentin pathway.

## Discussion

Given the poor survival rate of patients with metastatic RCC using conventional therapeutic strategies, treatments for metastatic RCC are urgently needed. Expression profiling is essential for the identification of novel tumor suppressive and oncogenic circRNAs, as well as to elucidate their mechanistic functions 16,34,35. Herein, we screened for circRNAs that are differentially expressed between metastatic RCC and matched tissues by microarray and focused on the underlying mechanism of increased circPRRC2A expression in RCC. We also established that the upregulation of circPRRC2A is likely caused by the downregulation of DHX9 in RCC, but not caused by the transcriptional inhibition of QKI or ADAR1. The upregulation of circPRRC2A in RCC tissues was significantly associated with larger tumor size and poor prognosis of RCC, indicating that circPRRC2A functions as an oncogene and might be associated with tumor growth and progression of RCC. Gain-of-function and loss-of-function experiments demonstrated that circPRRC2A is associated with cell invasion and metastasis. Thus, despite tumor heterogeneity, the tumor promoting role of circPRRC2A in EMT progression and angiogenesis was strongly and consistently supported by clinical data and in both *in vitro* and *in vivo* studies.

The ceRNA hypothesis posits that RNA transcripts, such as circRNAs, lincRNAs, and mRNAs share miRNAs response elements, and compete for binding to these miRNAs, thereby regulating the expression of each other. The interplay between these RNA molecules constructs a complex post-transcriptional regulatory network 36-40. The first circRNA that was confirmed to be a miRNA sponge was ciRS-7 which contains more than 70 binding sites for miR-7. Competitive inhibition of miR-7 by ciRS-7 increases the expression of oncogenes, consequently promoting the initiation and development of cervical cancer 41. Since ciRS-7 contains so many mir-7 binding sites, it can rapidly bind or release a great quantity of miR-7 molecules, thereby effectively regulating the network of diseases. Relative to targeting single oncogenes, inhibiting the expression of ciRS-7 could provide the advantage of affecting the activities of multiple oncogenes 42. Considering the abnormal expression abundance of circRNAs in cancer and various miRNA binding sites within mRNAs and circRNAs transcripts, sponging multiple miRNAs by single circRNA could be a common mechanism in the dysregulation of cancer cells 19,43,44.

Using bioinformatics analyses, we revealed miRNAs (miR-514a-5p/miR-6776-5p) that share common binding sites with circPRRC2A and TRPM3. We demonstrate using FISH that circPRRC2A and miR-514a-5p/miR-6776-5p show significant colocalization in the cytoplasm 45. To further establish that circPRRC2A functions as a ceRNA in the miR-514a-5p/miR-6776-5p/TRPM3 axis, we found that miR-514a-5p and miR-6776-5p reduced the activity of circPRRC2A luciferase reporter by at least 66% and 55%, respectively. Furthermore, we unveiled that TRPM3 was a common target of miR-514a-5p and miR-6776-5p. Subsequent biotinylated miRNA pull-down assays confirmed the competitive binding activities of circPRRC2A and TRPM3 to miR-514a-5p/ miR-6776-5p. Moreover, a rescue experiment showed that circPRRC2A significantly attenuated the effects of miR-514a-5p/miR-6776-5p on TRPM3. Concisely, circPRRC2A exerts its function as a ceRNA to competitively sponge miR-514a-5p and miR-6776-5p to abolish the endogenous suppression of TRPM3 by miR-514a-5p/miR-6776-5p. Upregulated expression of circPRRC2A lead to persistent activation of TRPM3-induced EMT signaling in RCC, suggesting that circPRRC2A may enhance EMT-promoted RCC metastasis by blocking miR-514a-5p/miR-6776-5p activity. Finally, we analyzed the regulatory pathways in which circPRRC2A may be involved by RNA-seq. GSEA demonstrated that sh-circPRRC2A resulted in a TRPM3-mediated EMT, cell adhesion and angiogenesis are the prominent causes of tumor cell metastasis. These data, collectively, suggests a critical role of circPRRC2A-sponging of miR-514a-5p/ miR-6776-5p in the control of TRPM3-induced EMT and invasiveness by means of a "one-hit/ multiple-targets" mechanism.

TRPM3, a member of the melastatin-like subfamily of TRP channels 46, stimulation induces an intracellular signaling cascade due to the increase in intracellular Ca^2+^ 47-49. Functionally, stimulating TRPM3 is linked to insulin secretion by pancreatic beta-cells, modulation of neurotransmitter release, and tumorigenicity 50. Maria FC *et al.* demonstrated that TRPM3 depletion reduced RCC tumorigenicity with only 3 small tumors formed (19%) relative to control cells that formed tumors in 100% of mice. Here, we showed that TRPM3 was upregulated in larger implantation tumor tissues and knockdown of circPRRC2A suppressed the expression of TRPM3. Importantly, elevated expression of TRPM3 in RCC is significantly associated with larger tumor size, higher TNM stage and poor prognosis, coincident with high expression of circPRRC2A in larger tumors and more severe TNM stages (Table S2).

As shown in Figure 7G, this study reveals that circPRRC2A may promote angiogenesis and tumor metastasis, and may provide prognostic biomarkers in patients with RCC. Moreover, the regulatory role of the DHX9-circPRRC2A-miR-514a-5p/miR-6776-5p- TRPM3 pathway was confirmed in RCC. Our results provide novel insight into understanding the development and progression of RCC and potential improved therapeutic strategies for RCC.

## Supplementary Material

Supplementary methods, figures, and tables 1-2.

Supplementary table 3.

Supplementary table 4.

Supplementary table 5.

Supplementary table 6.

## Abbreviations

RCC: Renal cell carcinoma
mRCC: Metastasis renal cell carcinoma
circPRRC2A: circular RNA PRRC2A
cDNA: complementary DNA
EMT: Epithelial-Mesenchymal Transition
IHC: Immunohistochemical
qRT-PCR: Quantitative real-time PCR
TCGA: The Cancer Genome Atlas
ncRNA: non- coding RNA
OS: Overall survival
MFS: Metastasis- free survival
RIP: RNA immunoprecipitation
shRNA: short hairpin RNA
WT: wild-type
MUT: Mutant-type
DHX9: DExH-Box Helicase 9
ADAR: Adenosine Deaminase RNA Specific
QKI: KH Domain Containing RNA Binding
