# Supplementary material for: circPRRC2A promotes angiogenesis and metastasis through epithelial-mesenchymal transition and upregulates TRPM3 in renal cell carcinoma
Source: Theranostics. 2020 Mar 15;10(10):4395–409. doi: 10.7150/thno.43239 (PMC7150475; doi:10.7150/thno.43239)
Supplement: Supplementary file 1 — Supplementary methods, figures, and tables 1-2. [file thnov10p4395s1.pdf]

Supplementary Information

circPRRC2A promotes angiogenesis and metastasis through epithelial-mesenchymal transition and upregulate TRPM3 in renal cell carcinoma

Wei Li[1,2], Feng-Qiang Yang[1,3], Chen-Min Sun[4], Jian-Hua Huang[1], Hai-Min Zhang[1], Xue Li[5], Guang-Chun Wang[1], Ning Zhang[2], Jian-Ping Che[1], Wen-Tao Zhang[1], Yang Yan[1], Xu-Dong Yao[1], Bo Peng[1], Jun-Hua Zheng[6] and Min Liu[7]

[1]Department of Urology, Shanghai Tenth People's Hospital, Tongji University, Shanghai, P. R. China.

[2]Department of Medical Oncology, Dana-Farber Cancer Institute, Harvard Medical School, Boston, MA, USA.

[3]Department of Urology, Ninghai Hospital, Branch of Shanghai Tenth People's Hospital, Zhejiang, P. R. China.

[4]Department of Anesthesiology, Tongren Hospital, Shanghai JiaoTong University School of Medicine, Shanghai, P. R. China.

[5]Department of Pathology, Beijing Chao-Yang Hospital, Capital Medical University, Beijing, P. R. China.

[6]Department of Urology, Shanghai First People's Hospital, Shanghai JiaoTong University School of Medicine, Shanghai, P. R. China.

[7]Department of Urology, Tongren Hospital, Shanghai JiaoTong University School of Medicine, Shanghai, P. R. China.

Supplementary Methods

Microarray analysis

In this study, microarray-based profiling was performed from 3 lung metastatic tissue samples from 3 patients with renal cell carcinoma (RCC). For controls, matched 3 renal carcinoma samples from 3 patients with RCC were used. The sample preparation and microarray hybridization were performed based on the Arraystar's standard protocols. After being digested with Rnase R (Epicentre Technologies, Madison, USA) to remove linear RNAs, circular RNAs were amplified and transcribed into fluorescent circRNA utilizing Arraystar Super RNA Labeling Kit (Arraystar V2). Subsequently, the labeled circRNAs were hybridized onto the Arraystar Human circRNA Array V2 (8×15K, Arraystar). Agilent Feature Extraction software (version 11.0.1.1) was used to analyze acquired array images. Quantile normalization and subsequent data processing were performed with using the GeneSpring GX v12.1 software package (Agilent Technologies). CircRNAs showing fold changes ≥ 2 and *P* values < 0.05 were regarded as significantly differentially expressed.

Cell lines and cell culture

The human RCC cell lines (ACHN, Caki-1, Caki-2 and 786-o) and Human umbilical vein endothelial cells (HUVECs) were all provided by the American Type Culture Collection (ATCC, Manassas, USA). A-498, 769-P and A-704 were purchased from Cell Bank of the Chinese Academy of Sciences (Shanghai, China). Human embryonic kidney epithelial cell line (HEK-293t) and HK-2 cell

line was kindly provided by Dr. Xiangmin Lv from Boston Children's Hospital, Harvard Medical School, Boston, MA, 02115 USA. 786-o and 769-P were cultured in RPMI-1640 Medium (Gibco, Grand Island, New York, USA) containing 10% heat-inactivated fetal bovine serum (HI-FBS, Hyclone, Logan, Utah, USA), 100 units/ml penicillin, 100 μg/ml streptomycin and 2 mM L-glutamine. ACHN, A-704 and A-498 cells were grown in Eagle's Minimum Essential Medium (EMEM, Gibco) with 10% HI-FBS, antibiotics and L-glutamine. HK-2 cells were grown in Dulbecco's Modified Eagle Medium: Nutrient Mixture F-12 (DMEM/F12) plus 1% keratinocyte growth supplement (KGS, Thermo Fisher Scientific, USA) with 10% HI-FBS and antibiotics. HEK-293t cells were grown in Dulbecco's Modified Eagle's Medium (DMEM) with 10% HI-FBS and antibiotics. Caki-1 and Caki-2 cells were grown in McCoy's 5A Medium (Gibco) with 10% HI-FBS, antibiotics and L-glutamine. HUVECs were cultured in Vascular Cell Basal Medium (GIBCO) containing 10% HI-FBS, endothelial cell growth supplement (ECGS, 0.03 mg/mL) (Biomedical Technologies, MA, USA). All cells were negatively tested for mycoplasma contamination and authenticated based on STR fingerprinting before use.

Plasmid, miRNA mimics/inhibitors, Transient transfection and construction of stable RCC cell lines

Plasmid mediated circPRRC2A overexpression and knockdown vector were obtained from IBSBIO (Shanghai, China), the full-length cDNA of human circPRRC2A was synthesized by Invitrogen and cloned into the pCDNA3.1

expression mini vector (Supplementary Fig. 1a, c). shRNA targeting circPRRC2A were obtained from GenePharma (Shanghai, China). miR-514a-5p and miR-6776-5p mimics/inhibitors and control plasmid were ordered from IBSBIO. All sequences used are listed in Supplementary Table 1a. For RCC cell transfection, cells were seeded in 6-well plates and were cultured to 70%-80% confluence before transfection. According to the manufacturer's instructions, miRNA mimics, inhibitors or corresponding controls (IBSBIO) were transiently transfected using Lipofectamine 3000 (Invitrogen, Carlsbad, California, USA).

For lentiviral transduction, virus-containing supernatant was collected 48 h after the cotransfection of packaging plasmids and the shRNA- or circRNA-overexpressing vector into HEK-293t cells, followed by its addition to the target cells. The stable cell lines were then infected by miRNAs and corresponding controls according to the manufacturer’s instructions. 48 hours later, the infected cells were selected with 2 μg/ml of puromycin (Gibco, USA).

Biotin coupled miRNAs capture

The biotin-coupled miRNA pull-down assay was performed as described previously16,23. Briefly, the 3’-end biotinylated miRNA-RNA mimic or control biotin-RNA was transfected into RCC cells at final concentration of 25 nmol/L for 24 h. The biotin-coupled RNA complex was then pulled down by incubating cell lysates with streptavidin-coated magnetic beads (IBSBIO). miRNA and TRPM3 levels in bound fractions were evaluated by qRT-PCR.

Western blot assay

Total proteins from cells were extracted using RIPA lysis buffer (Thermo Fisher, USA) and protein concentrations were measured with BCA protein assay (Pierce, IL, USA). Protein bands were visualized by ECL (Thermo Fisher). Western blot analyses were performed according to standard protocols as described previously. The antibodies used are listed in Supplementary Table 1d.

Actinomycin D and RNase R treatment

Transcription was interfered with the addition of 2 μg/ml Actinomycin D (Sigma-Aldrich, USA) at indicated time point. Total RNA (3 μg) was incubated for 15 min at 37°C with 3 U/μg of RNase R (Lucigen Technologies, WI, USA). After treatment with Actinomycin D or RNase R, the RNA expression levels of circPRRC2A and linar-PRRC2A were analyzed by qRT-PCR.

Cell proliferation and colony formation assay

Cell proliferation was examined using the Cell Counting Kit (CCK)-8 assay (Abcam, ab228554, USA). 1×10^3 cells were seeded in 100 μl of complete culture media in 96-well plates for various time periods. CCK-8 assay was performed to measure cell viability according to manufacturer's instructions. For colony scoring, the cells were fixed in 1 ml of methanol for 15 min and were stained with crystal violet (Sigma-Aldrich, USA) for 20 min. The numbers of colonies were counted and expressed as mean ± SD of three independent experiments.

Wound healing and cell invasion assay

5×10^5 cells were cultured in 6-well plate until confluence and then

wounded with a 20 μl pipette tip. Migration photos were captured at 0, 24 h and 48 h after scratching. Transwell assays using Boyden chambers containing 24-well Transwell plates (Sigma-Aldrich, CLS3460-48EA) with 8 mm pore size were used to evaluate the migration and invasiveness of cells. All experiments were performed in duplicate and repeated three times.

RNA fluorescence in situ hybridization (FISH)

The FISH assay was performed in ACHN/Caki-1 cells as previously described. Biotin-labelled probes specific to circPRRC2A and Dig-labelled miR-514a-5p and miR-6776-5p probes were used in the hybridization. The sequence is listed in Supplementary Table 1b, c. The signals of biotin-labelled probes were detected using Cy5-Streptavidin (Life Technologies). The signals of Dig-labelled miR-514a-5p and miR-6776-5p probes were detected using a tyramide-conjugated Alexa 488 fluorochrome TSA kit. Nuclei were counterstained with 4,6-diamidino-2-phenylindole. Images were acquired on a Leica confocal microscope (Leica Microsystems, Germany).

Immunofluorescence

HUVEC cells grown on the confocal dish (Corning) were rinsed three times with PBS and fixed with 4% paraformaldehyde for 15 min. After washing with PBS, cells were permeabilized with 0.1% TritonX-100 in PBS for 10 min on ice. Cells were then washed three times with PBS, blocked with 5% BSA in TBST for 30 min at 37 °C and incubated for 1 h at 37°C with primary antibodies. After washing with TBST, cells were incubated with corresponding secondary

antibody for 1 h at 37°C. HUVECs were then stained with DAPI for nucleus staining. The relative mean fluorescence densities were analyzed by *Image J*, and plotted using GraphPad Prism 5.0 software.

Luciferase reporter assay

The sequence of circPRRC2A was cloned downstream of Luci-reporter vector (IBSBIO). Mutations were performed in the binding sites. Luci-reporter vector was cotransfected with the predicted miRNAs or miR-NC into 293t or RCC cells by Lipofectamine-mediated gene transfer. The relative luciferase activity was normalized to Renilla luciferase activity 48 hours after transfection.

RNA immunoprecipitation (RIP)

RIP assay was performed by using a Magna RIP RNA-Binding Protein Immunoprecipitation Kit (Millipore) according to the manufacturer's instructions. The antibodies were used as follows: anti-Flag (Anti-FLAG® M2 Antibody, Cell Signaling Technology, USA), anti-IgG (ab186733, Abcam, USA).

Immunohistochemistry (IHC) staining

The experimental methods are based on previous study. For evaluation of IHC staining, a semiquantitative scoring criterion was used, in which both staining intensity and positive areas were recorded. A staining index (scores 0-12), obtained as the intensity of DHX9, Ki-67 or PRRC2A positive staining (negative =0, weak =1, moderate =2, or strong =3 scores) and the proportion of immunopositive cells of interest (<10% =1, 10%-50% =2, 50%-80% =3, ≥80% =4 scores), was calculated. The following primary antibodies were listed in

Supplementary Table 1d.

***In vivo* metastasis mice model**

All procedures involving mouse were approved by the Institutional Animal Care and Use Committee of Shanghai Tenth People’s Hospital of Tongji University. All mice were housed in laminar flow cabinets under specific pathogen-free conditions at room temperature with a 12 h light/dark cycle, with food and water available ad libitum. To investigate the effects of circPRRC2A on tumor formation *in vivo*, we designed two mice models: one is an subcutaneous xenotransplanted tumor model, and the other is in vivo metastasis mouse model. First, ACHN and Caki-1 cells stably transfected with sh-circPRRC2A or control cells (8.0×10^6 cells/site) were injected subcutaneously into 4 weeks old female BALB/c nude mice. The developing tumors were observed during the following 8 weeks, after which the mice were sacrificed. The weights of each tumor were also documented. Tumor volume was calculated as $V = \text{length} \times (\text{width}^2)/2$. *In vivo* metastasis model was established by intravenous injection of 1.0×10^6 RCC cells through the tail vein into each 4 weeks old female BALB/c nude mice. After 40 days, six mice in each group were killed, and their lungs and livers were resected and then partly fixed in 10% buffered formalin. The numbers of pulmonary metastatic nodules in the lung/liver were observed.

Supplementary Figures and Legends

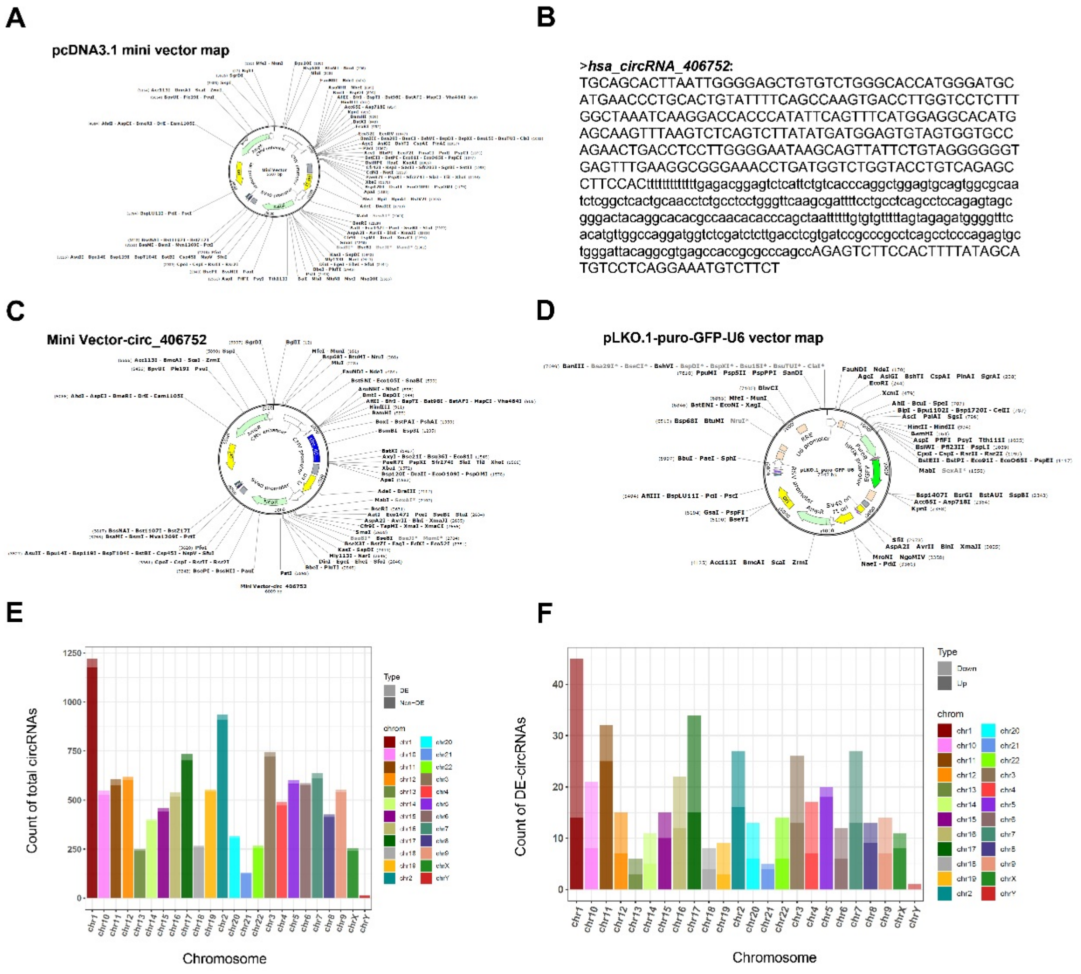

Supplementary Figure 1. circPRRC2A overexpression vector and circRNA expression profile in RCC. **A** circPRRC2A sequence was constructed into pcDNA3.1 mini-circ vector **C**. **B** Full sequence information of circPRRC2A. **D** sh-circPRRC2A plasmid pLKO.1-puro vector. **E** The chromosomal distribution of total circRNAs. The upper light color is differential expression circRNAs. **F** The chromosomal distribution of tatal 387 differentially expressed circRNAs. The upper light color is down-regulation expression of circRNAs.

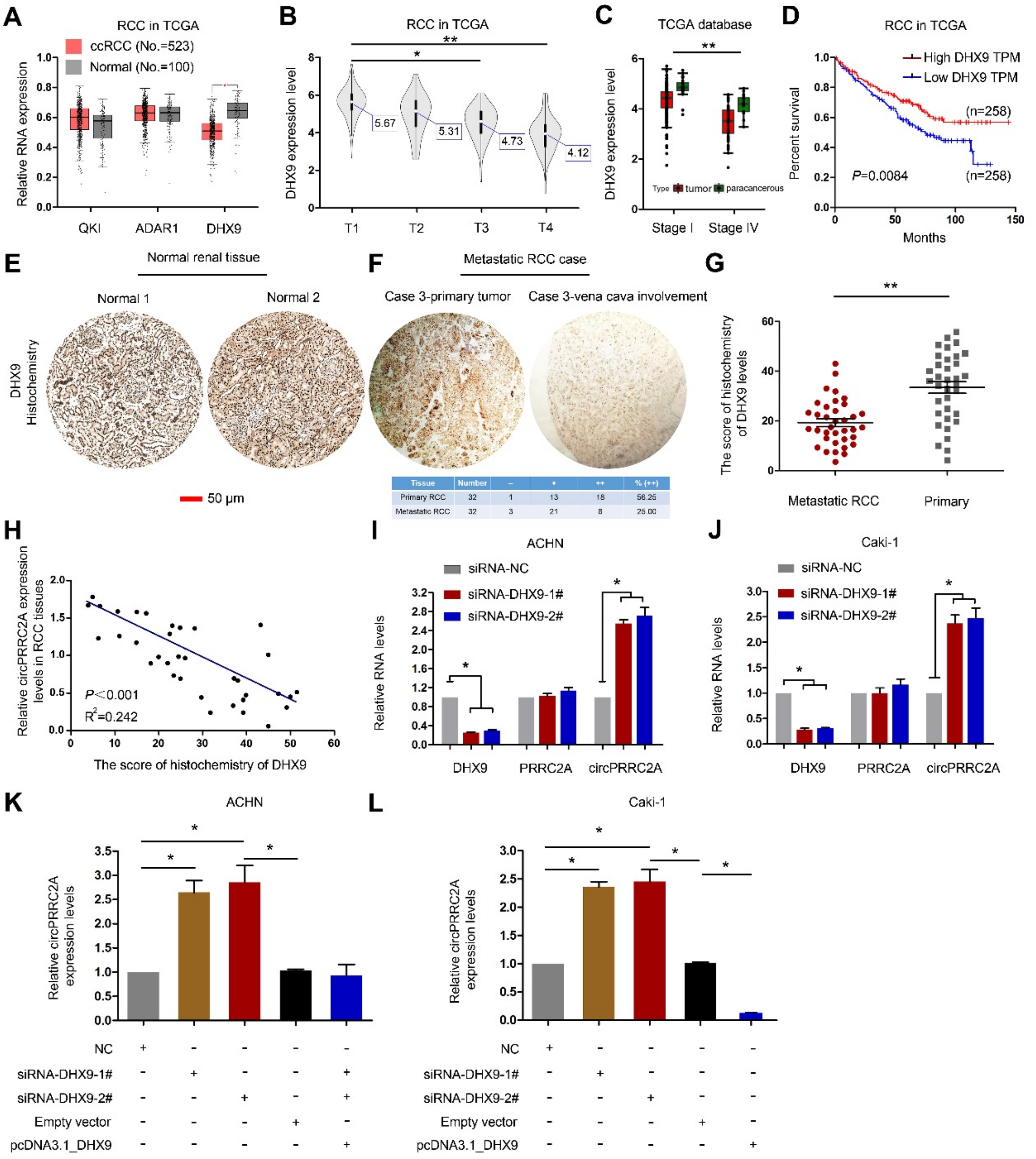

Supplementary Figure 2. The level of circPRRC2A is associated with DHX9 expression in RCC. **A** The TCGA database revealed the mRNA expression of DHX9 was significantly down-regulated in RCC tissues. The boxplot analysis showed log2 (TPM + 1) on a log-scale. **B**-**C** The expression level of DHX9 in RCC patients with different tumor T-stages. **D** Kaplan-Meier's survival curves depicted the correlations between DHX9 and OS of RCC patients from TCGA database, the patients were stratified into two groups according to the median of DHX9. Log-rank test was used. **E** Immunohistochemical stains of DHX9 in renal normal tissues. **F** IHC staining and **G** scoring of DHX9 in 32 RCC primary tissues and vena cava involvement tissues. **H** The correlation between the IHC score of DHX9 and the relative expression of circPRRC2A in 32 RCC tissues. The correlation was measured by Pearson correlation analysis (R^2 = 0.242). **I**

qRT-PCR for DHX9, PRRC2A and circPRRC2A upon DHX9 depletion using DHX9-siRNA in ACHN cell lines, **J** Caki-1 cell lines. **K**-**L** DHX9 significantly reduces expression of circPRRC2A, which can be alleviated with DHX9 (siRNA). DExH-Box Helicase 9, DHX9; adenosine deaminase 1 acting on RNA, ADAR1; Quaking, QKI. Data indicate mean ± SD, n = 3; *P < 0.05, **P < 0.01.

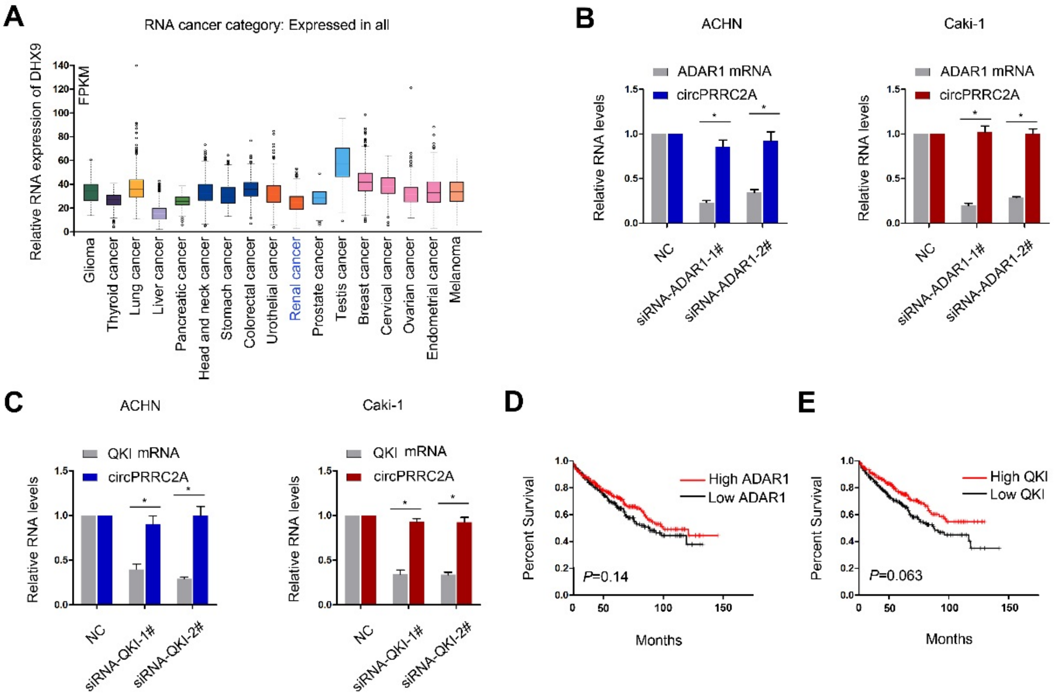

Supplementary Figure 3. circPRRC2A is regulated by DHX9 instead of ADAR1 and QKI. **A** The expression levels of DHX9 in RNA cancer category (TCGA database). **B** and **C** qRT-PCR showed that circPRRC2A mRNA did not show significant changes after knocking down ADAR1 or QKI. **D** Kaplan-Meier's analyses of correlations between ADAR1 or QKI level at OS of RCC patients (log-rank test). All data are presented as means ± SD. **P* < 0.05.

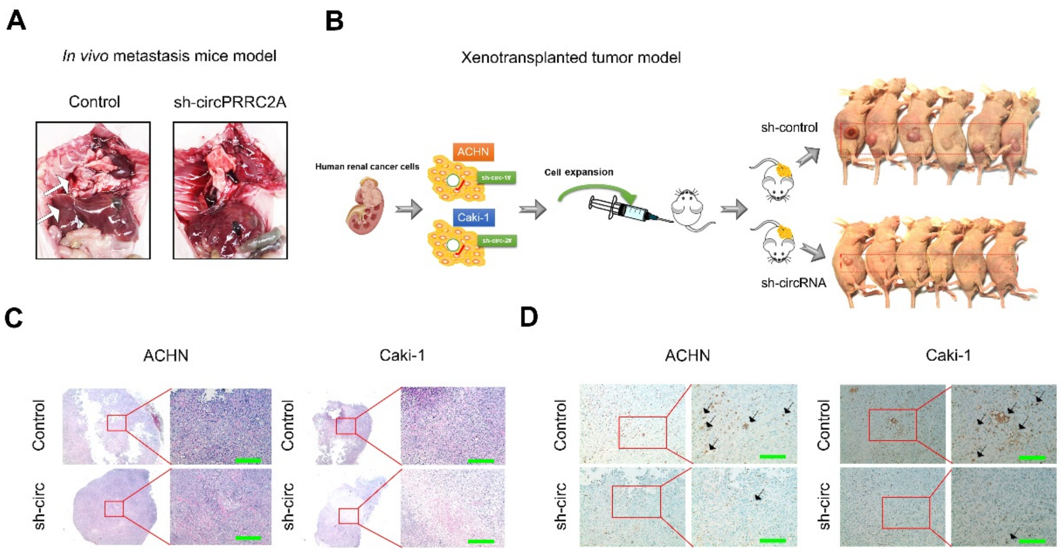

Supplementary Figure 4. **A** Stably transfected RCC cells with sh-circPRRC2A or sh-control with their respective control lentivirus were injected into the caudal vein of BALB/c nude mice for 8 weeks. Representative images of lungs, metastatic nodules (indicated by white arrows) are shown. **B and C** Representative pictures of HE staining in xenograft tumor model tissue sections at three magnifications. **D** The expression of Ki-67 was decreased in circPRRC2A down-regulated expression group when compared with control group. Bars represent for 100 μm, 20× and 50 μm, 40×.

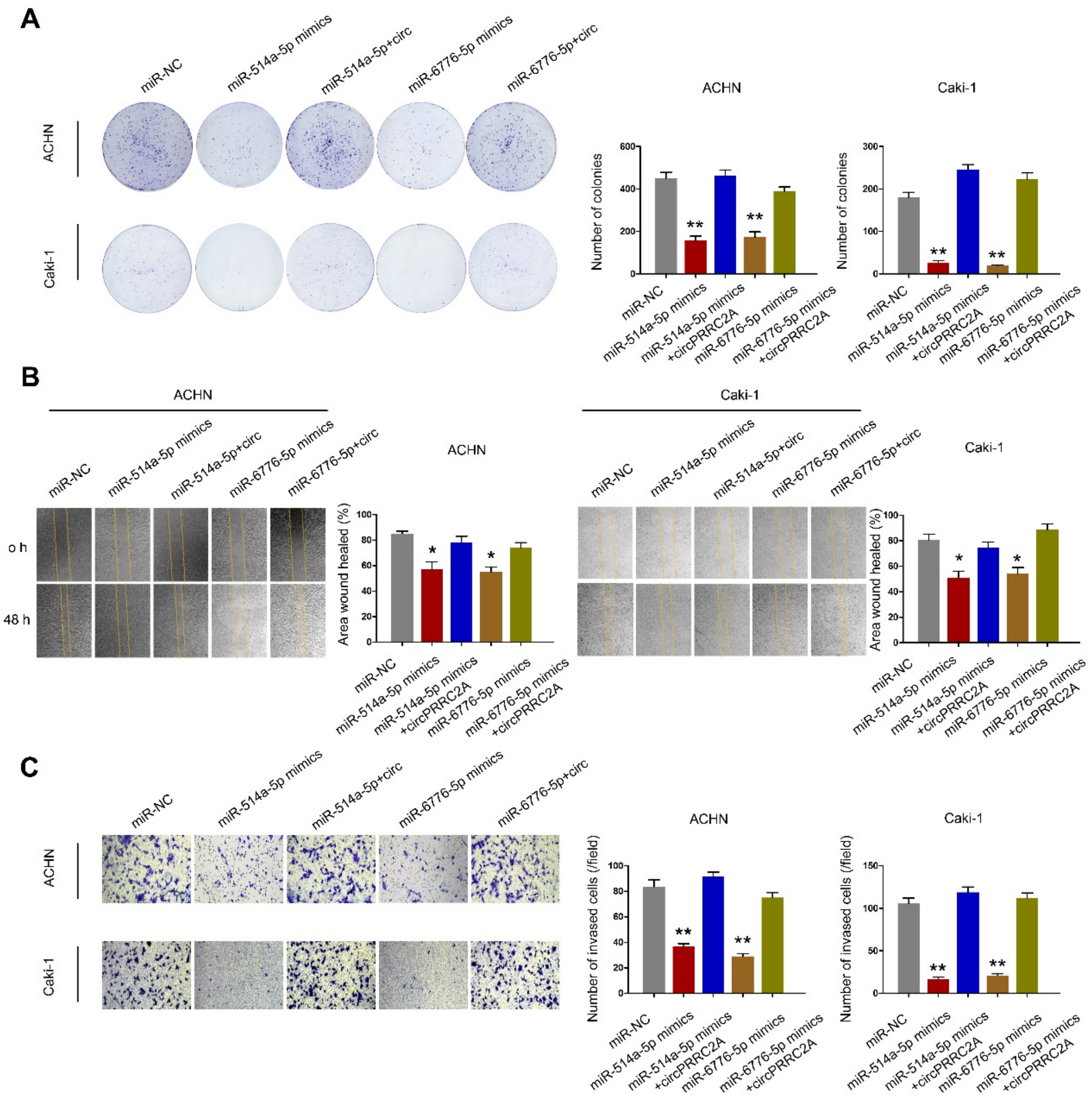

Supplementary Figure 5. miR-514a-5p and miR-6776-5p promotes proliferation, migration and invasion of RCC cells. **A** miR-514a-5p or miR-6776-5p can inhibit the cell clonality of RCC cells measured by colony formation assay, and the suppression was retarded after over-expression of circPRRC2A. **B** miR-514a-5p or miR-6776-5p can suppress the migration of RCC cells measured by wound healing assay, and the suppression was retarded after over-expression of circPRRC2A. **C** miR-514a-5p or miR-6776-5p suppresses the metastasis of RCC cells, as evidenced by transwell assay, and the suppression was retarded after over-expression of circPRRC2A. Data indicate mean ± SD of three experiments. **P* < 0.05, ***P* < 0.01.

Table S1. Characteristics of patients with ccRCC and associations with clinicopathologic variables.

Variable	Total (%)	Variable	Total (%)
**Gender**		**Fuhrman grade**	
Male	91 (77.1)	1-2	67 (56.8)
Female	27 (22.9)	3	39 (33.1)
**Age at surgery (years)**		4	12 (10.1)
≤45	12 (10.2)	**AJCC clinical stage**	
＞45，≤55	70 (59.3)	I	36 (30.5)
＞55	36 (30.5)	II	33 (28.0)
**BMI**		III	27 (22.9)
＜25	66 (55.9)	IV	22 (18.6)
≥25	52 (44.1)	**Metastatic status**	
**Maximum tumor size (cm)**		NM	69 (58.5)
≤4	30 (25.4)	LM	34 (28.8)
＞4，≤7	43 (36.4)	DM	15 (12.7)
＞7，≤10	34 (28.8)	**T stage**	
＞10	11 (9.4)	T1	36 (30.5)
**Renal capsule invasion**		T2	31 (26.3)
No	69 (58.5)	T3	29 (24.6)
Yes	49 (41.5)	T4	22 (18.6)

Abbreviation: BMI=body mass index; DM=tumours involving distant metastases; LM=tumours involving lymphatic metastases; NM=tumours involving non-metastasis; ccRCC=clear cell renal cell carcinoma.

Table S2. Univariate and multivariate model measured ORs and 95% CIs of ccRCC associated with distant and other covariates.

Variables (No.)	Tumor metastasis (%)	Univariate		Multivariate	
		p	OR	*p*	OR (95% CI)
**Gender**		0.817	0.538	0.423	0.574 (0.312-1.631)
Male (91)	35 (29.7)				
Female (27)	14 (11.9)				

**Age at surgery**		0.619	0.942	0.531	1.826 (1.189-2.823)
≤55 (82)	29 (24.6)				
＞55 (36)	20 (16.9)				
**Maximum tumor size**		0.051	3.518	0.037*	2.014 (1.312-4.198)
≤7 (73)	18 (15.3)				
＞7 (45)	31 (26.3)				
**Fuhrman grade**		0.032*	1.829	0.026*	2.732 (1.163-4.326)
1-2 (67)	15 (12.7)				
3-4 (51)	34 (28.8)				
**pT Stage**		0.009*	4.132	0.013*	3.780 (1.693-5.715)
T1-T2 (67)	8 (6.8)				
T3-T4 (51)	41 (34.7)				
**CircPRRC2A expression**		0.001*	5.373	0.009*	4.132 (1.709-6.264)
Low (23)	6 (5.1)				
High (95)	43 (36.4)				
**TRPM3 expression**		0.007*	3.917	0.021*	3.650 (1.172-6.172)
Low (31)	11 (9.3)				
High (87)	38 (32.2)				

Abbreviation: CI=confidence interval; Tumor metastasis= tumours involving distant metastases and/or lymphatic metastases; OR=odds ratio; ccRCC= clear cell renal cell carcinoma.

*Statistically significant.
